# Confirmation of successful supraglottic airway device placement in neonates using a respiratory function monitor

**DOI:** 10.1038/s41390-025-03810-x

**Published:** 2025-02-05

**Authors:** Robyn Dvorsky, Tobias Werther, Katharina Bibl, Michael Schneider, Christoph Binder, Lisa Habrina, Katrin Klebermaß-Schrehof, Veronika Kranebitter, Georg M. Schmölzer, Angelika Berger, Michael Wagner

**Affiliations:** 1 Division of Neonatology, Pediatric Intensive Care and Neuropediatrics, Department of Pediatrics, Comprehensive Center for Pediatrics, Medical University of Vienna, Vienna, Austria; 2 Division of Phoniatrics and Speech-Language Therapy, Department of Otorhinolaryngology, Head and Neck Surgery, Medical University of Vienna, Vienna, Austria; 3 Centre for the Studies of Asphyxia and Resuscitation, Neonatal Research Unit, Royal Alexandra Hospital, Edmonton, AB Canada; 4 Department of Pediatrics, University of Alberta, Edmonton, AB Canada

## Abstract

**Background:**

This study investigated the use of a respiratory function monitor (RFM) to guide the placement of a supraglottic airway device (SAD) in neonates during intensive care interventions. We hypothesized that using a RFM would decrease the number of attempts needed for a successful placement.

**Methods:**

This single-center pilot study was carried out at a tertiary NICU at the Medical University of Vienna. Patients were ventilated using a SAD during neurosurgical or endoscopic interventions. A RFM was either hidden (but recording) or visible to providers during SAD placement. Feedback from the RFM was used to assess correct/incorrect placement and optimize ventilation quality. The parameter leakage was used for assessment: if leak was <30%, correct placement was assumed. The primary outcome was the number of attempts until correct placement. Secondary outcomes included ventilation parameters recorded by the RFM and the duration of SAD placement.

**Results:**

Six patients were included in this pilot trial. Using a RFM to guide SAD placement led to fewer attempts (median attempts: 3 [hidden] vs. 1 [visible]). Furthermore, using the RFM, necessary adaptations were made to the SAD position to decrease leakage (mean leakage: 74.8% [hidden] vs. 17.8% [visible]), subsequently endoscopy after insertion of SAD using the RFM then confirmed anatomically correct position.

**Conclusion:**

This pilot study indicated that a RFM might be useful to provide guidance during SAD placement.

**Impact statement:**

- Feedback from a RFM reliably indicated correct anatomical placement of a SAD by correlating low leakage values with proper SAD positioning.
- RFM guidance could improve neonatal airway management, reducing procedural time and number of attempts.
- We present promising preliminary results. Further research is needed to confirm these findings.

## Introduction

Supraglottic airway devices (SADs) are airway management tools that are routinely used in adult medical care and their application is now gaining increasing popularity within neonatal care.

The European Resuscitation Council recommends the use of a SAD in the newborn life support algorithm as an alternative to mask ventilation.^1,2^ The SAD might address two common problems of neonatal resuscitation: Firstly, ventilations via face mask are not always effective, especially in the case of significant mask leakage.^3^ Secondly, even though endotracheal intubation is a highly effective and advanced method for establishing a secure airway, it is a difficult skill to master and maintain and should only be performed by trained specialists due to the risk of complications and high failure rates.^4^ Therefore, the SAD could be a compromise between these two methods, as it enables more effective ventilations with less leakage and less need for secondary intubation, and its placement is a skill that might be considerably easier to acquire than endotracheal intubation. Previous studies showed that manikin-based simulation training is a valid option for training of SAD placement leading to higher levels of confidence in the performance of this skill among trainees. The reported duration of SAD placement in these studies was short (5–12 s on average) and success rates were high (no failed first attempts).^5–7^ Thus, this skill should be accessible to a larger number of providers.

A review by Schmölzer et al. evaluated SADs in the setting of neonatal resuscitation and concluded that SADs constitute a safe alternative to bag-mask ventilation in infants > 34 weeks of gestational age and of > 1500 g.^8^ Incidentally, the authors of a 2017 review on the use of the SAD in neonatal resuscitations noted that research efforts were limited in patients < 34 weeks of gestation or < 1500g birth weight.^9^ The majority of SADs currently available are only licensed for infants above 2000g. However, there have been case reports demonstrating that SAD placement is possible even in infants weighing between 800 g to 900g.^10,11^

A recent meta-analysis by Diggikar et al. discussed the comparison of SADs with bag-mask ventilation and endotracheal intubation in low- to middle-income countries. They could show that during neonatal resuscitations, the SAD was significantly better (risk ratio [RR] = 0.23) than the face mask for performing positive pressure ventilations (PPV) in regard to ventilation failure rate (i.e., failure with primary device) and the subsequent need for intubation. Furthermore, they also identified that time to spontaneous breathing (mean difference 4.4 s) as well as ventilation times (mean difference 20.1 s) were significantly shorter for patients that were ventilated using the SAD.^12^

A respiratory function monitor (RFM) measures and displays ventilation parameters (expiratory tidal volume [V_Te_], peak inspiratory pressure [PIP], mask leak, ventilation rate, positive end expiratory pressure [PEEP]) in real-time.^13^ This enables airway providers to evaluate the quality of their ventilations and change their technique, if necessary.^13^ Until now, respiratory function monitoring has been used primarily during mask ventilation or for intubated patients. Several previous simulation-based studies^14,15^ and clinical trials^16–18^ have shown improvements in ventilation quality when a RFM was available during PPV. However, there have been hardly any research efforts evaluating the combined use of a SAD and a RFM.

This pilot study evaluated a novel approach to airway management, which is the application of a RFM to guide SAD placement. Furthermore, we provide subsequent endoscopy video recordings demonstrating the anatomical placement of the SAD for three patients.

## Methods

### Study setting

The single-center pilot study was carried out at a tertiary NICU at the Medical University of Vienna.

### Hypothesis

We hypothesized that the use of a RFM will support neonatal healthcare providers during the placement of a SAD and that the number of attempts for correct placement can be decreased.

### Study design

Patient recruitment and data collection initially occurred as part of a larger prospective intervention trial, where all patients receiving any form of manual positive pressure ventilation were included.^13^ This study was registered on ClinicalTrials (ID NCT05512689) and was reviewed by the institutional review board of the Medical University of Vienna (number: 1334/2022).

However, due to comparability issues (i.e., different ventilation device [SAD], addition of bronchoscopy) these six patients were later omitted from the original trial, and data recorded from these patients was separately analyzed for this pilot study. The local institutional review board has also granted a positive votum on the amendment addressing this retrospective data analysis (number: 1334/2022).

**Fig. 1:**
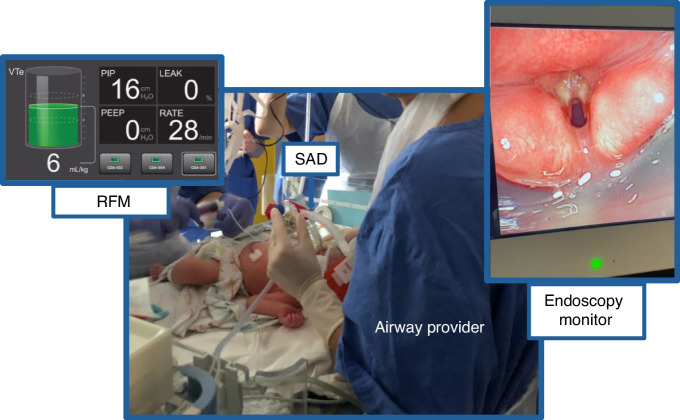
Study setup. Study setup with provider placing the SAD and using the RFM for guidance (on the left). On the right side an example of the endoscopic view into the neonate’s larynx through the SAD and view of the position of the laryngeal mask above/around the larynx (as indicated with the black arrows) is shown.

Due to the design of the original study, the first two patients in this secondary analysis received PPV via the SAD with a hidden RFM. The following four patients were ventilated with a RFM that was visible during PPV to provide feedback on ventilation parameters during placement and for ongoing ventilations. The RFM (Neo100, Monivent, Gothenburg, Sweden) measured and recorded five ventilation parameters (i.e., expiratory tidal volume [V_Te_], mask leakage, peak inspiratory pressure [PIP], ventilation rate, positive end-expiratory pressure [PEEP]) using a flow sensor, which wirelessly transmitted to a monitor in real-time each ventilation. During and after SAD (iGel size 1, Intersurgical, Wokingham, UK) placement, clinicians with the visible RFM were able to review these ventilation parameters and adapt SAD position accordingly (see Fig. 1 for study setup). Correct placement of the SAD was assessed using the parameter “leak”. Correct placement of the SAD was assumed if leakage on the RFM was low ( < 30%). If values for leakage were above 30%, incorrect placement was assumed and adaptations to the SAD position were made (i.e., adjusting depth and angle of the SAD). The value of 30%, however, was not a strict cut-off, but rather used as a guide for airway providers. We are aware that there is no universally defined range, but we have defined a leak >50% as excessive in the original trial.^13^ Likewise, leakage of <30% seems sensible enough to evaluate successful placement. Previous trials have used the same.^19,20^

We tested if placement using the RFM was successful using endoscopy (bronchoscopy), which showed the anatomical placement of the SAD. Four of the included patients received bronchoscopy solely based on clinical indication/as a diagnostic tool, therefore, patients had to be sedated and the airway had to be secured, for which we used a SAD. The obtained videos were then used for evaluation of the SAD placement. Bronchoscopy was performed by the local otorhinolaryngology staff or a specially trained neonatologist (MW). The bronchoscope (XP190 rhinolaryngoscope, Olympus Corporation, Tokio, Japan) was inserted through the SAD and if SAD placement was successful, the larynx was visible at the end of the SAD through the camera at the tip of the endoscope (see supplementary material, videos 1–3).

If the RFM was not available during SAD placement (in case of the first two included patients), successful placement was assessed using clinical parameters (chest excursion, rise in heart rate and oxygen saturation, auscultation). If these parameters were not satisfactory (i.e., no rise in heart rate), adaptations were made to the SAD position and, finally, if this was not successful, the placement attempt was stopped, and manual ventilation was performed before another attempt was commenced.

### Subjects

There were no exclusion criteria for patients regarding weight, gestational age, or comorbidities. We did not match cases and controls. For enrollment in this study, patients had to receive an intervention at the NICU requiring sedation and therefore some form of airway management. Informed consent was obtained from legal guardians of all included patients within this study. Providers were neonatal fellows and consultants working at the NICU.

### Measures

The primary outcome in this study was the number of attempts needed for successful placement of the SAD. Secondary outcomes were derived from RFM recordings during and after placement of the SAD. Vital parameters during the procedures are also reported. We provide three video recordings from bronchoscopies in the supplemental material. Video recordings of the interventions were obtained and used to determine important events as well as to match RFM data to the clinical situation (i.e., if adaptations were made), but will not be shared.

### Statistical analysis

Data recorded by the RFM was analyzed using mean, standard deviation, median and interquartile range depending on normality of distribution. No further statistical testing was performed due to the small number of patients. The duration of SAD placement and the number of attempts were analyzed using video recordings of the interventions.

## Results

A total of six patients were included in this pilot study, of these, one patient was female and five were male. Patients weighed between 1140 g and 4405 g at the time of intervention. Patient age at intervention ranged from 30 + 5 weeks of gestational age to 19 months (see Table 1). All infants received ventilatory support using the iGel SAD size 1. Four SADs were inserted by fellows and two by neonatal consultants. Fellows were in their first, second and third year of specialty training, consultants had about ten years of clinical experience. Participating physicians did not have specific training with the SAD prior to use.

**Table 1:** Patient characteristics.

	sex	weight at intervention (birth weight)	GA at intervention (GA at birth)	intervention	indication	provider
**patient 1**^**a**^	m	1450 g (1574 g)	34 + 1 (29 + 2)	neurosurgical	posthemorrhagic hydrocephalus	consultant
**patient 2**^**a**^	f	4405 g (3055 g)	19 months (37 + 3)	bronchoscopy	surfactant-dysfunction syndrome	resident
**patient 3**^**ab**^	m	3192 g (2730 g)	59 + 0 (38 + 2)	bronchoscopy	esophagotracheal fistula	resident
**patient 4**^**ab**^	m	3180 g (3260 g)	43 + 3 (36 + 3)	bronchoscopy	tracheomalacia	resident
**patient 5**^**a**^	m	1140 g (630 g)	30 + 5 (23 + 3)	neurosurgical	posthemorrhagic hydrocephalus	resident
**patient 6**^**ab**^	m	3912 g (2100 g)	6 months (n.r.)	bronchoscopy	suspected subglottic stenosis	consultant

*GA* Gestational age.

^a^iGel size 1 was used for all patients.

^b^endoscopy videos obtained for these patients.

n.r. = not recorded for this patient.

Two out of six patients (patients 1 & 2) were ventilated using a SAD and a hidden RFM. In case of the other four patients, the RFM was visible to providers and could be used to assess correct placement as well as to adjust SAD position and ventilation technique once inserted (see Table 2 for all RFM data).

**Table 2:** Metric outcome parameters.

		leakage *%*	pV_Te_ *%*	VTe_mean_ *ml/kg*	PIP *cmH*_*2*_*O*	rate *vent/min*	attempts *number*	duration^a^ *seconds*	
**patient 1**	before placement	93.25	0.00	1.69	27.34	26.21	4	16 (79)	RFM hidden
	after placement	5.08	35	8.18	21.78	32.95			
**patient 2**	after placement	56.29	29.37	3.68	17.19	34.44	2	12 (29)	
**patient 3**	before adaptations^b^	75.57	100	5.90	17.10	22.17	1	12	RFM visible
	after adaptations^b^	18.83	92	6.05	13.11	21.2			
**patient 4**	before adaptations^b^	49.13	0.00	1.04	25.31	40.71	1	19	
	after adaptations^b^	16.42	39.4	7.67	27.82	35.86			
**patient 5**	before adaptations^b^	31.22	0.00	1.35	20.23	34.83	1	31	
	after adaptations^b^	28.61	72.73	4.88	19.78	29.79			
**patient 6**	after placement	7.26	66.7	5.92	23.77	24.61	1	20	

*pVTe* tidal volume percentage in range, *VTe* tidal volume, *PIP* peak inspiratory pressure,

^a^at successful attempt (total, i.e. all attempts summated)

^b^adaptations (repositioning/direction, deeper insertion) based on RFM data.

Regarding the primary outcome parameter, we observed that fewer attempts were necessary to achieve correct placement when providers had visual access to the RFM (median: 1 attempt in visible group vs. 3 attempts in hidden group).

Duration of placement per attempt did not differ significantly between patients. The mean time for placement at successful attempts was 18 (12–31) seconds, unsuccessful attempts lasted 18.5 (14–22) seconds on average.

By reviewing the RFM data, we were able to identify that the mean leakage during PPV after first insertion of the SAD was 93.3% for patient 1 and 56.3% for patient 2, where the RFM was not visible to providers, suggesting inadequate placement of the SAD and ineffective ventilations. In comparison, mean leakage for patients 3 to 6, where the RFM was visible to airway providers and respective adjustments (i.e., reposition of SAD) could be made, was lower (median: 17.6%). Ventilatory data from patients 3 to 5 also suggested that initially high leakages could be detected using the RFM and subsequently be reduced through optimization of SAD position during the intervention, i.e. improvements in this value after adaptations were made (patient 3: 75.6% to 18.8%, patient 4: 49.1% to 16.4%, patient 5: 31.2% to 28.6%).

Through endoscopy, we were able to verify that correct placement of the SAD around the larynx coincided with low values for leakage displayed on the RFM.

Four patients underwent bronchoscopy and were ventilated manually (patients 2 – 4 and 6) using the SAD. They were breathing spontaneously shortly after the procedure was completed and the SAD could be removed. Mechanical ventilation was only necessary in the two patients who underwent neurosurgical intervention (patients 1 and 5), which lasted 1.25 and 4 h, respectively.

## Discussion

In this study, we report pilot data on the use of an RFM to guide SAD placement and SAD ventilation in six neonates. This pilot trial showed that with a visible RFM, less attempts are needed to achieve successful SAD placement.

This study might have several implications for the clinical use of SADs in preterm and term neonates. To our knowledge, the use of laryngoscopy to evaluate SAD placement within neonates has not been studied before, especially in the context of validating data from a RFM to accurately predict inadequate or correct SAD placement.

While the SAD is generally an airway management tool that is easy to use, the difficulty in the neonatal patient collective is achieving the exact right placement. Due to the complex airway anatomy and small size of these patients, often very subtle adaptations (i.e., a slight shift/angling forward or a slightly deeper position of the SAD) are required to achieve a satisfactory position. The RFM can be a great help to minimize the time until adaptations are made, if adaptations are necessary, but can also help to avoid unnecessary adaptations and even unnecessary additional placement attempts. It can be hypothesized that it is more time efficient as well as gentle for patients to perform slight adaptations to SAD position instead of initiating a new placement attempt. We do not yet know the impact of multiple SAD placement attempts on the occurrence of adverse events but based on data from endotracheal intubations (i.e., approximately 25% of providers needing ≥ 3 attempts, with an adverse event rate of >40%),^21^ we could expect to see a similar relationship. Regarding patient 1, a total of four attempts were needed to achieve correct placement of the SAD. By reviewing the video recording of this intervention, it was evident, that the application of a SAD in a very small patient has its limitations. Even two very experienced neonatal consultants would have benefitted from additional feedback or information, other than clinical, supporting correct SAD placement or necessary adjustments. This highlights the relevance of combining the SAD with respiratory function monitoring, especially if used on preterm infants. To underline this point, we would like to discuss patient 5, who weighed even less than patient 1 at the time of intervention (1140 g). In this case, the RFM was visible to providers and only one placement attempt was needed, even though the patient weighed less and the provider was less experienced (resident vs. consultant).

There is limited evidence regarding the use of SADs in patients <1500 g. Some authors have reported on the use of a SAD in this patient collective (Brimacombe: 800g,^10^ Trevisanuto: 880g^11^ Pinheiro: 1150g,^22^ Smee: 1200g,^23^ Wanous: 1290g,^24^ Barbosa: 1335g,^25^ Brimacombe: 1360g,^26^ Micaglio: 1530g^27^). All authors observed that SAD placement in infants of this weight was feasible and improved respiratory status, coinciding with our results.

Wanous et al. investigated the use of a SAD for surfactant administration (i.e., SALSA, Surfactant Administration Through Laryngeal or Supraglottic Airways). They found that placement could be achieved in less than 35 s in 65% of patients, which is in line with our results (providers with visible RFM required between 12 and 31 s). Furthermore, they observed that vital parameters remained stable during SAD placement,^24^ which was also observed in our patient collective (see Table 3). SALSA is an interesting new field of application for SADs,^28,29^ a RFM could potentially be applied in this setting as well to ensure correct placement of the SAD before the instillation of surfactant into the neonates’ airways.

**Table 3:** Vital parameters.

	SpO_2_ (%)	HR (bpm)	RR_syst_ (mmHg)	RR_diast_ (mmHg)	RR_mean_ (mmHg)
**patient 1**	Max. 98	Max. 189	Max. 80	Max. 45	Max. 58
	Min. 96	Min. 168	Min. 52	Min. 26	Min. 38
	Mean 97	Mean 177	Mean 67	Mean 36	Mean 48
**patient 2**	Max. 100	Max. 147	Max. 103	Max. 72	Max. 84
	Min. 90	Min. 113	Min. 103	Min. 68	Min. 82
	Mean 95	Mean 130	Mean 103	Mean 70	Mean 83
**patient 3**	Max. 99	Max. 144	Max. 80	Max. 49	Max. 60
	Min. 88	Min. 122	Min. 66	Min. 34	Min. 46
	Mean 95	Mean 131	Mean 73	Mean 41	Mean 53
**patient 4**	Max. 100	Max. 140	Max. 65	Max. 39	Max. 54
	Min. 97	Min. 127	Min. 52	Min. 31	Min. 38
	Mean 99	Mean 134	Mean 58	Mean 34	Mean 45
**patient 5**	Max. 100	Max. 144	Max. 78	Max. 47	Max. 61
	Min. 90	Min. 127	Min. 59	Min. 29	Min. 44
	Mean 96	Mean 137	Mean 65	Mean 40	Mean 51
**patient 6**	Max. 100	Max. 154	Max. 84	Max. 56	Max. 67
	Min. 98	Min. 129	Min. 65	Min. 34	Min. 45
	Mean 100	Mean 140	Mean 72	Mean 43	Mean 54

*SpO*_*2*_ oxygen saturation from pulse oximetry, *HR* heart rate, *RR*_*syst*_ systolic blood pressure, *RR*_*diast*_ diastolic blood pressure, *RR*_*mean*_ mean arterial pressure.

A trial by Pejovic et al. investigated differences in respiratory parameters (leak and tidal volume) between SAD and face mask using a hidden RFM. They did not observe differences in respiratory parameters, though the SAD group had a quicker return to normal values for heart rate (time to heart rate >100bpm: 13 (9–15) seconds with SAD vs. 61 (33–140) seconds with face mask ventilation, *p* = 0.0002).^30^

In this study, we used video recordings of bronchoscopy in four out of the six included patients to demonstrate that the RFM, as a non-invasive monitoring tool, can reliably predict correct SAD position. We wanted to point out, that we do not suggest the use of endoscopy as a general method to check SAD placement (patients in this trial received endoscopy solely based on clinical need). Previous reports on airway management during endoscopy in pediatric patients showed that the use of a SAD is feasible for this purpose.^31,32^ Plessis et al. used fiberoptic endoscopy to determine SAD position in adult patients.^33^ In another study, the authors used fiberoptic endoscopy in pediatric patients to determine SAD position and visibility of the larynx, depending on the size of the SAD. They found that smaller sizes more frequently showed less optimal anatomical placement and may have to be repositioned more often.^34^ This might also be a common problem in the extremely low birth weight neonates, as there are currently no available SADs with sizes smaller than size 1, which might not be appropriate for infants weighing < 800 g.

Furthermore, we want to point out that even though this study was carried out during neonatal intensive care interventions in the NICU, we speculate that the results are also applicable to neonatal resuscitations in the delivery room. In this trial, we have shown that the parameter “leakage” provided by the RFM can be used as surrogate marker to evaluate correct SAD placement (as validated with endoscopy). These findings should also hold true in resuscitation scenarios, although this hypothesis will have to be tested in future trials. The SAD is a safe, easy and quick method to secure the airway, which might be even more important during highly stressful newborn resuscitations. The RFM can be connected easily and swiftly. Therefore, the combined use of a RFM and a SAD seems well suited for emergency settings.

With the possibility to obtain reliable ventilation data, it becomes possible to assess for correct placement of the SAD and therefore give assurance to providers, subsequently increasing the safety of this airway management procedure.

It is of note, that data in the current trial is derived from only six patients and findings are therefore merely observational and should be interpreted with caution. Our results prompt the possibility for future research into this method of airway management in the form of prospective randomized trials.

### Strengths and limitations

The main strength of this study is the report of an innovative new approach to respiratory management in the NICU using the combination of RFM and SAD placement. The integration of endoscopy video recordings to substantiate the accuracy of RFM data to assess SAD placement validates these findings.

A limitation of this study is the small number of patients. We are aware that prospective trials with a higher number of participant/patients are necessary, however we provide this promising preliminary data to generate new ideas for future prospective research. Comparability between the six patients included in this study is limited, as they had varying weights, gestational ages, and comorbidities.

## Conclusion

This pilot trial provides preliminary results indicating that with the use of a RFM to guide SAD placement, the number of attempts until successful placement can be decreased. With this combined use of SAD and RFM, we introduce a new approach for the use of SADs in neonatal patients with the potential to improve patient safety during airway management interventions. While the patient number was small, our findings are promising and lay the foundation for further evaluation in prospective clinical trials.

## Supplementary information

Video 1

Video 2

Video 3

## Data Availability

The datasets generated during and/or analyzed during the current study are available from the corresponding author on reasonable request.
